# Efficacy of Probiotics in Management of Celiac Disease

**DOI:** 10.7759/cureus.22031

**Published:** 2022-02-08

**Authors:** Basit Ali, Ali Raza Khan

**Affiliations:** 1 Medicine, Nishtar Medical Univeristy, Multan, PAK; 2 Medicine, Nishtar Medical University, Multan, USA

**Keywords:** diarrhea reduction, gluten-free diet, microbiota, probiotics, celiac disease

## Abstract

Objective

The objective of this study was to interpret any rule of probiotics in the management of celiac disease and apply the results to improve the quality of life of patients with celiac disease if the result comes in favor of probiotics.

Materials and methods

It was a cross-sectional study conducted in the gastroenterology unit of Nishtar Medical University, Multan. A total of 170 children with celiac disease were enrolled in the study and divided into two groups (A and B) using a computer-generated table of random numbers. Group A was given only a gluten-free diet, while group B was given probiotics and a gluten-free diet. The efficacy of probiotics was measured in terms of reduction in stool frequency at the end of the 28 days of treatment. The data was recorded on the datasheet for every individual, and the statical analysis was performed using the Chi-square test. The patients were fully explained about the research purpose, and written consent was taken from them.

Results

The efficacy of probiotics in children with celiac disease was compared in both groups. Results showed a marked reduction in the frequency of stools to less than half, i.e., 90.59% (n=77) in group B and 63.53% (n=54) in group A. The Chi-Square test resulted in a p-value of 0.000027 showing a significant difference in both groups.

Conclusion

Probiotics are found to be highly efficient in terms of reduction in diarrhea in celiac disease. Probiotics will improve not only quality of life but also play an essential role in managing celiac disease.

## Introduction

Celiac disease (CD) is a gluten-sensitive enteropathy, triggering an immune response to gluten ingestion [1]. It affects up to one percent of the population [2]. Gluten is mainly found in wheat flour, rye, barley, and oats [3]. Children with CD classically present with abdominal distension, diarrhea, and failure to thrive [4]. Adults mainly present with chronic diarrhea and bloating [5]. Extraintestinal manifestations include anemia, fatigue, arthritis, infertility, liver failure, neuropathy, schizophrenia, or autism [6].

CD being multifactorial depends on genetic, immunological, and environmental factors. Histologically it is characterized by total or partial atrophy of the intestinal villi resulting in low absorption of nutrients [7,8]. The primary genetic risk factors include human leukocyte antigen (HLA)-DQ8/DQ2 haplotypes [9]. Genetic predisposition alone is not sufficient, and additional environmental factors are always required for disease development. Although gluten proteins have significant contributions to environmental factors, recent studies have suggested that gut microbiota shifts may also contribute to gluten sensitivity [10,11].

The microbiota, the microorganisms that live in or out of the human body, has a crucial role in the immune system's maturation and in developing protective/tolerogenic immune responses [12]. Several life events may prime a dysregulated gut microbiota, starting from a delivery mode: increased risk in C-section newborn, breastfeeding, infectious agents, and antibiotic intake [13-16]. The majority of microbiota consists of *Lactobacillus*, *Prevotella*, and *Bifidobacteria*. To date, the only effective treatment option is a gluten-free diet [17]. Probiotics have been implied as a treatment strategy considering the role of microbiota in disease pathogenesis [18,19]. Probiotics are nonpathogenic live organisms and, when administered orally in adequate amounts, alter the host's microflora and help in digestion and inhibit the bacterial colony in the gut, causing disease, including *Lactobacillus rhamnosus*, *Bifidobacterium lactis*, *Bifidobacterium infantis*, *Saccharomyces boulardii*, and Lactobacillus plantarum, etc. [20].

The study's motive was to recce the probiotics' role in reducing diarrhea in CD patients considering its action mechanism compared to a gluten-free diet alone.

## Materials and methods

Study location

This study was conducted in the gastroenterology department of Nishtar Nishtar Medical University, Multan, Pakistan, after approval from the Institutional Review Board which is licensed under the health ministry of the Government of Pakistan.

Sample population

Children in the age range from 8 to 10 years who had recently been diagnosed with celiac disease on the basis of the intestinal biopsy were included in this descriptive cross-sectional study. They were also undergoing treatment for their disease. However, children with other causes of diarrhea like cystic fibrosis, chronic amebiasis, chronic giardiasis, and severe malnutrition were excluded. A total of 170 children were selected for this study.

Data collection

Patients meeting the inclusion and the exclusion criteria were selected using a computer-generated table of random numbers. They were decided into two groups - group A and group B.

A comprehensive history and clinical examination of the patients were made. The frequency of stools per day was noted significantly. Group A was given only the gluten-free diet. Group B was started on Gutcare™sachet 500mg (*Clostridium butyricum* and *Bifidobacterium*) diluted in 75-100ml of boiled water twice a day for 28 days, in addition to the gluten-free diet. After 28 days, patients were reviewed for the number of stools per day. We defined efficacy as the reduction of stool frequency to more than half after the given treatment. Data were collected using specially designed charts. To ensure follow-up and to avoid any bias, contact numbers and addresses of the patients were taken.

Statistical analysis

Data were expressed as frequencies and percentages. The quantitative variables like frequency of stools per day were presented as mean and standard deviation. A Chi-square test was applied to compare the two groups. A p-value of less than 0.05 was considered to show a significant effect.

Ethical considerations

A written consent stating the purpose, methods, risks, benefits, and assurance of data's confidentiality was taken from all patients' parents/guardians.

## Results

A total of 170 cases (85 in each group) were included in the study to determine the efficacy of probiotics in children with CD by comparing them with the control group. There was no significant difference among groups regarding age and gender, so these variables are not discussed. The mean number of stools per day before and treatment is shown in Table 1.

**Table 1:** Mean number of stools per day

	Group A (n=85)		Group B (n=85)	
	Mean	Standard deviation	Mean	Standard deviation
Before treatment	5.19	1.1	4.67	0.8
After treatment	3.8	1.4	1.81	0.94

The efficacy of probiotics in children with celiac disease was compared in both groups. Results showed a marked reduction in the frequency of stools to less than half, i.e., 90.59% (n=77) in group B and 63.53% (n=54) in group A. The Chi-Square test resulted in a p-value of 0.000027, showing a significant difference in both groups.

**Table 2:** Efficacy in two groups

	Group A (n=85)		Group B (n=85)	
	Frequency	Percentage	Frequency	Percentage
Effective	54	63.53	77	90.59
Not effective	31	36.47	08	9.41

## Discussion

The advantageous effects of probiotics in the treatment of CD can be postulated due to the following reasons: (a) they produce toxins like bacteriocins against the pathogens; (b) they block the binding sites of gut microbiota and also compete for their nutrition; (c) they regulate the body's immune responses [21]. The current study was planned to investigate probiotics' role in reducing diarrhea in patients of the CD based on its mechanism of action compared to a gluten-free diet alone. This study contains several potential limitations. The study mainly evaluates the role of probiotics in reducing diarrhea while excluding the non-diarrheal and atypical presentations of CD. The reason for the exclusion was the lack of quantifiable variables to measure the outcome in such patients. According to some studies in Pakistan, many people range between 20.7% [22] to 48.1% [4] present with atypical CD symptoms.

Our study shows that probiotics effectively reduce diarrhea with a gluten-free diet compared to a gluten-free diet alone. These results agree with a similar randomized control trial conducted at the Department of Pediatrics, DHQ Allied Hospital Faisalabad [23]. Probiotics considerably studied in CD are *Lactobacilli *and *Bifidobacterium *species. Most of the evidence on the effect of probiotics in CD comes from animal models. Some mouse models demonstrate immunomodulatory effects of probiotics and reduced gliadin-induced inflammation [24-26].

Studies regarding probiotics and CD in humans are very scarce. In a recent randomized control trial, Olivares et al. demonstrated that the administration of *Bifidobacterium longum *for three months in children newly diagnosed with CD, when associated with the gluten-free diet, significantly reduced the number of *Bacteroides fragilis* and secretory IgA in the stool [27]. The results of the study support the result of the present study. 

Results of our study also honor some other studies demonstrating the immunomodulating action of probiotics as a beneficial effect. Klemenak et al. investigated the effects of two *Bifidobacterium breve* strains on serum interleukin-10 and tumor necrosis factor-alpha (TNF-alpha) levels in children with CD on the gluten-free diet, demonstrating after three months treatment reduction in the level of TNF-alpha while interleukin-10 level remained unchanged [28].

The role of probiotics in promoting a potential role in microbiome restoration can also reduce CD symptoms like diarrhea. In 2018, Primec et al. showed that a probiotic mixture of two *Bifidobacterium breve *strains modulated acetic acid production and total short-chain fatty acids, resulting in microbiome restoration [29]. Some studies also showed that probiotics' beneficial effects were due to digesting or altering gluten polypeptides. De Angelis et al. analyzed the specific probiotics' role containing *Bifidobacterium *and *Lactobacillus *species in hydrolyzing the gliadin polypeptides [30]. Interestingly, another study by De Angelis et al. reported that a single probiotic strain is insufficient to degrade gliadin peptides and, therefore, must be used together with other strains [31]. These results follow the usage of a mixture of probiotics in our study. 

Recent studies also demonstrated that probiotics for the CD with a gluten-free diet are necessary to reduce the damage caused by a gluten-free diet alone. De Palma G et al. found that a gluten-free diet reduces the colonization of healthy bacteria, including *Lactobacilli *and *Bifidobacterium *species, while promoting harmful gram-negative bacterial growth such as *E. coli* and *Enterobacteriaceae* [32]. Moreover, a gluten-free diet may be rich in high glycemic index foods, increasing insulin resistance and the risk of obesity and cardiovascular diseases. 

At this time, the only effective treatment for CD is a lifelong gluten-free diet. Complete gluten withdrawal is challenging to achieve because even small amounts of gluten are harmful. Moreover, adherence to a diet is difficult for many patients. There is a high variation in compliance with therapy. Data showed that 80% of people diagnosed before four years of age are adherent to their diet, while those diagnosed after four years of age are only 40% compliant [33]. This adds the rationale for using probiotics combined with a gluten-free diet to improve compliance or replace a gluten-free diet.

The study had some limitations too. Firstly, it involved a mere comparison among two groups, i.e., one using the gluten-free diet and the other using probiotics in addition. No placebo control was used due to a lack of available resources and financial constraints. This might have created a bias in the results produced. Secondly, no other variables except stool frequency were studied. Thirdly, minor side effects like abdominal bloating and flatulence were considered insignificant in interpreting the results. Fourthly, the dose of probiotics was given the same in all patients regardless of their age, weight, and height.

## Conclusions

Probiotics have proved to have beneficial roles in diseases involving the gut, urogenital tract, skin, and respiratory tract. Our study focused on their role in patients with celiac disease, and they were found to be highly efficient in reducing diarrhea in celiac disease. They will improve not only the quality of life but also play an essential role in managing celiac disease.
